# Body mass index & low CIR in colonoscopy! 

**Published:** 2018

**Authors:** Adnan Qureshi, Saira BiBi, Ravi Madhotra

**Affiliations:** *Department of Surgery and Gastroenterology, Milton Keynes University Hospital NHS Trust. Standing Way, Eaglestone, Milton Keynes Buckinghamshire MK6 5LD, UK *

**Keywords:** BMI, Colonoscopy, Cancer

## Abstract

**Aim:**

The aim of this study was to determine common factors leading to incomplete colonoscopy with a special interest in patient body mass index (BMI), and also to determine most common second line investigation, its pick up rates for cancer and the success rate of re-scoping.

**Background:**

Wide availability of scope guide in all procedures may decrease failure rate.

**Methods:**

We retrospectively reviewed 2891 colonoscopies performed at our institution from August 2015 to July 2016. The cohort was composed of all incomplete procedures (148) during this time period and a second cohort (148) of complete examinations which were randomly selected for relation of BMI only. The data in incomplete colonoscopy group included age, gender, BMI, causes of failure, mode of referral, second line investigation. The success of re-scope to pick up a cancer was compared to other modalities i.e. CT Colonography etc.

**Results:**

Male to female ratio was 1:4.8. High incomplete colonoscopy rate was noted in females (81%). Mean age in failure group was 64 ±15. Average BMI was 28± 15.Most common mode of referral was urgent or suspected cancer (74%). Common cause of failure was patient intolerance (30%). Most common anatomical site of failure was sigmoid colon (35%). Completion rate of re-scoping in experienced hands was 95%. A lower BMI is related with higher chances of failure or vice versa.

**Conclusion:**

Lower BMI has higher chance of failure, possibly due to less extra colonic fat leading to tortuous colon. Female sex is second most common cause of failure due to low intolerance to pain. Using stronger pain relief and equal distribution of these characteristics on different list will have least implications in busy cancer screening unit.

## Introduction

 Colorectal cancer is the second leading cause of cancer related death in the UK (1). In UK, the National Bowel Cancer Screening Programme involves biennial faecal occult blood testing (FOBT) with population participation of more than 50%. Whereas recently introduced “Bowel scope” program has the population participation of 39% (2,3). The participation for Bowel scope is increasing, and getting close to FOBT screening (4).This will increase the endoscopic workload tremendously in future. 

Because of increasing number of colonoscopies there is significant increase in risk and expense for the procedure. There is evidence that variability in its performance i.e. failure to perform complete test, can affect the outcome significantly. However, many factors increasing the risk of incomplete colonoscopy have previously been described including patient, endoscopist and instrument factors.

The British Society of Gastroenterology, the UK Joint Advisory Group (JAG) on GI Endoscopy, and the Association of Coloproctology of Great Britain and Ireland (ACPGBI) have developed quality assurance measures and key performance indicators for the delivery of a safe and affective colonoscopy within the UK. One of the key point indicator is caecal intubation rate, according to the guideline, colonoscopists should achieve 90% unadjusted caecal intubation rate (CIR).

Certain patient's factors, such as prior abdominal surgery or complicated diverticular disease, had been reported to hinder CIR during colonoscopy. Incomplete colonoscopies pose a clinical concern because management strategies to assess patients with incomplete colonoscopies vary from one center to another. In most of the centers, CT colonography (CTC) is second line investigation for a failed colonoscopy. 

We tried to review common causes of failure in our setting with special interest to patient’s Body mass Index (BMI) leading to incomplete colonoscopy. 

## Methods

The reports of all patients undergone colonoscopy from August 2015 to July 2016 at Milton Keynes University Hospital NHS Trust (MKUH) were reviewed. MKUH participate in bowel screening program for Bucks in southern hub and receive urgent 2ww referral for endoscopy.

MKUH endoscopy department performs approximately 3000 colonoscopies per year. Patients with incomplete colonoscopy were identified by reviewing computerized medical records of patients who underwent colonoscopy during this time interval.

The inclusion criteria was an incomplete colonoscopy. Exclusion criteria was age less than sixteen and people who were not suitable for colonoscopy and were cancelled on the day of colonoscopy. Bowel preparation was achieved using either MoviPrep (Poly Ethylene Glycol) or Picolax (Sodium Picosulphate). A standard 240 series Olympus (Olympus, USA) adult colonoscope was used for all cases initially. Patients were sedated with intravenous midazolam of 1-2 mg and i.v analgesia i.e. Fentanyl 50-100 μg or pethidine 25-50mg were titrated to patient comfort.

WHO check list and standard monitoring of patient’s heart rate and oxygen saturation were carried out. Routine manoeuvres i.e CO2 insufflation, and change of patient position were used, Infacol (simethicone) solution and or Buscopan (Hyosin) was used with caution to overcome discomfort due to bowel spasms in some cases.

 A standard list is 11 points in 4 hours session where each diagnostic colon carries 2 point giving adequate time of 40 mintues for each colonoscopy. In complete colonoscopies cecum was confirmed by identification of the ileocecal valve, appendicular orifices and triradiate fold.

Beside demography, indication, bowel prep, patient discomfort, BMI, reason for failure and further plan to complete visualisation of colon were all collected. Results are presented in descriptive statistics. Means and SDs are used to report continuous variables following a normal distribution, and median are used to report non-normal continuous variables. 

## Results

The failed colonoscopy data was collected from August 2015 to July 2016, using hospital electronic system. 

Total of 2891 colonoscopies were performed during this period, one hundred and forty-eight patients had failed colonoscopy with a failure rate of 5%.Out of one hundred and forty-eight colonoscopies only two were from screening program i.e 1.3%. The BMI of 2891 endoscopies were reviewed, where BMI was not documented in endoscopy admission paper work, weight and height measurements during last hospital visit were used to calculate the BMI, as long as it was with in last 6 months. The average BMI was 28 ± 15. A sample of hundred and forty-eight successful colonoscopies was randomly selected for BMI comparison only. Patients were divided in BMI groups to relate the outcome.

Failed colonoscopy group shows mean ±SD of age 64.3±15 years. Interestingly male to female ratio shows only 19 % were male and the rest 81% were female patients. The incomplete colonoscopies were shared between eight colorectal surgeons, eight gastroenterologist, including screening endoscopist, and two specialist endoscopy nurses **Fig 7**. Data also include the training list for colorectal and gastroenterology trainees supervised by trainers who have attended a training course and more than three year experience in endoscopy.

The highest rate of failure was in first year of qualifying as endoscopist, but interestingly the rate of failure become static after first year. Interesting ly, surgeons have slightly higher rate of failure. Although training endoscopies list are supervised directly by a trained experienced endoscopist but 9% of failed endoscopies were on training list which explain patient decreasing tolerance with length of procedure and pain.

**Figure 1:**
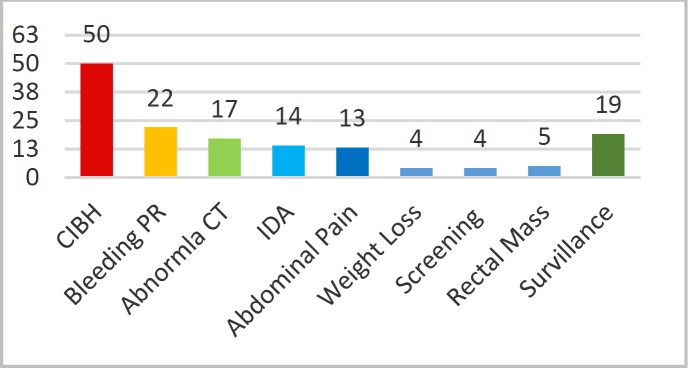
Indications for colonoscopy

The 86 % of colonoscopic evaluation were diagnostic. In the failed endoscopy group main indication for initial referral was” change in bowel habits” (CIBH) 33 %, “bleeding per rectum” 15%, and surveillance 14%, and the rest of indication are shown in Figure 1.

**Figure 2:**
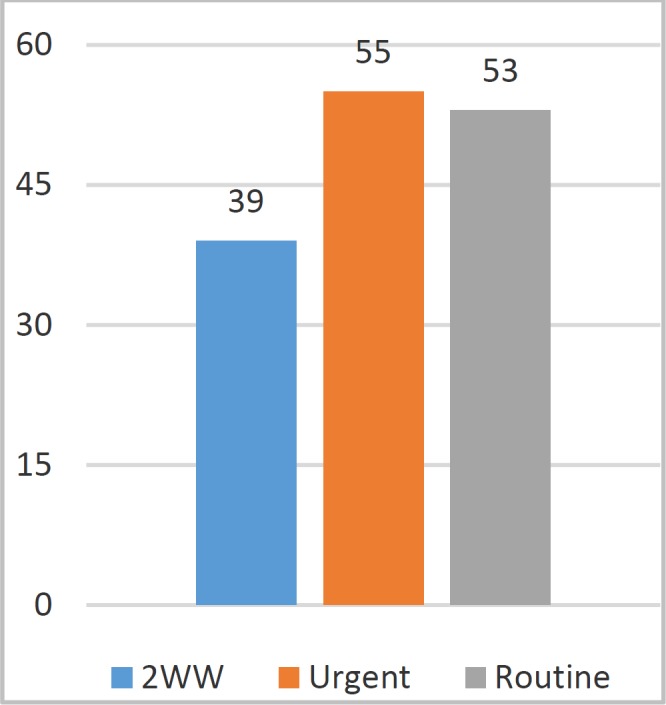
Mode of Referral

**Figure 3:**
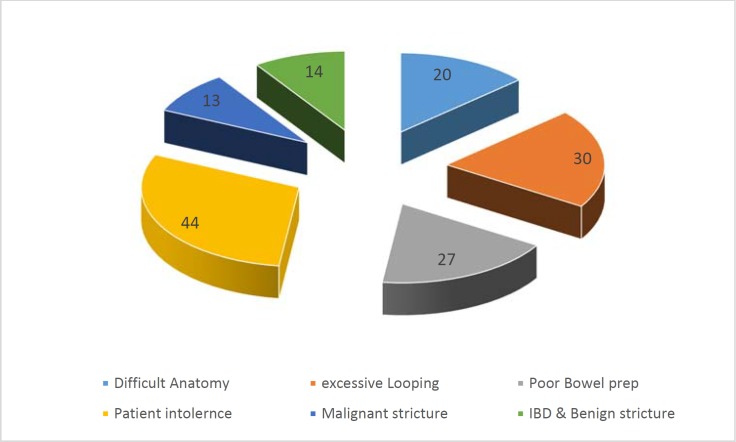
Causes of failure

Most common mode of referral was urgent i.e. two week wait (2ww) as shown in Figure 2. In this study main reasons for incomplete colonoscopy was patient intolerance (30%), looping (20%), poor bowel prep (18%), difficult anatomy (13.5 %), obstructing mass (10%) and benign stricturing disease (8.5%). (Figure 3).

**Figure 4:**
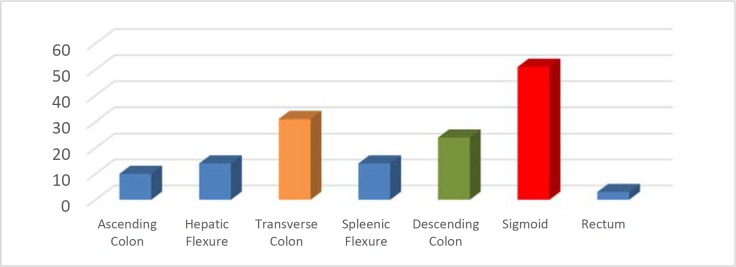
Common anatomical site of failure

**Figure 5:**
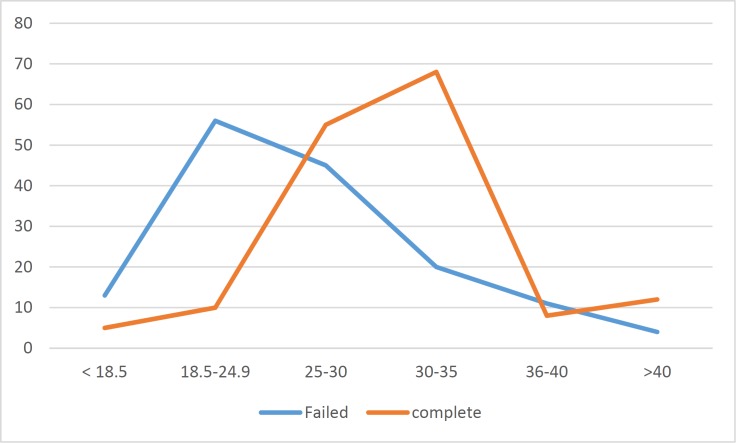
BMI relation

**Figure 6:**
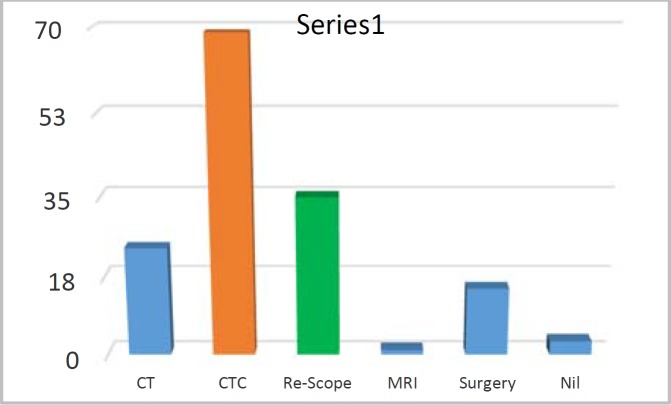
Second line investigation

Most common anatomical area of failure was sigmoid colon 34%. On the whole, left side of the large bowel was common site of failure i.e 62% Figure 4. Common reason for left sided failure is understandably due to propensity of sigmoid colon for looping, diverticular disease and due to common site for obstructing colonic neoplasms.

Interestingly, in failed endoscopy group 46 % had a BMI less than 25 compared to 23% who had BMI above 30. The incidence of failure was low with high BMI in this failed endoscopy group when compared to complete colonoscopy sample. (Figure 5). 

In failed colonoscopy group the bowel was visualized with second line investigation, most common was CTC (46%) (Figure 6). In CTC sub-group main indication was difficult anatomy at first endoscopy 92%. Polyps pick up rate was in 12 % (8) in CTC. No cancer was identified.

Repeat colonoscopy was second most common second line investigation 24% ordered with a completion rate of 95%. Interestingly, all these repeat colonoscopies were performed by endoscopist with more than 5 year experience in colonoscopy. All repeat colonoscopies were performed using routine maneuvers, patient positioning and a standard 240 Olympus colonoscope. 

A paediatric colonoscope was used in few cases only an over tube or an enteroscope was not used. No procedures were performed under fluoroscopy. No complications occurred during repeat colonoscopy. 

Repeat colonoscopy subgroup polyps pick up rate was 2% (1). In repeat colonoscopy sub-group main indication to repeat was poor bowel preparation 42% at first colonoscopy. Repeat colonoscopy failed in two patients which later on, found to have advanced colonic cancer on CT scan.

## Discussion

Incomplete colonoscopy not only poses a diagnostic dilemma for patients but also puts endoscopist under pressure to achieve minimum 90 % unadjusted caecal intubation rate according to JEG guideline. Over all the reported rate of incomplete colonoscopies ranges from 4% to 25% according to published literature (5- 8). Reasons for incomplete colonoscopy have been reported in literature and include difficult anatomy particularly sigmoid colon, marked diverticular disease, obstructing masses and strictures, angulation or fixation of colonic loops, adhesions due to previous surgery, spasm, poor colonic preparation, female sex or older age, and a low body mass index (9-11).

By comparing to literature, our result have demonstrated these finding again, lower BMI and female sex have a higher chances of colonoscopy failure, possibly due to loss of extra colonic fat and pelvic surgery. Increased incidence of pelvic surgery by mean age i.e 64, possibly due to hysterectomy, is a contributing factors.

Multiple techniques have been used to complete previous incomplete colonoscopies including use of a paediatric scope, gastroscope, external straightener (ie, overtube) and scope guide, all having varying degree of success (14-17).

After the introduction of CTC in 1994, it has gained wide speared acceptance as a best radiological means of detecting colonic cancer and polyps. It also help in detecting extra colonic abnormalities. CTC has sensitivity and specificity of above 90 % in detecting colorectal neoplasms, as reported in different studies (18, 19). In addition, several studies have shown CTC to be a valuable tool in evaluating the proximal colon after incomplete colonoscopy (20-25). 

A busy radiology department with limited capacity and high volume of patients for suspected cancer pathways limited the role of CTC, as first line investigation, in this study. Although there is no significant difference between CTC and colonoscopy in detection rate of colorectal cancer and large polyp, but still due to radiation exposure and therapeutic issues CTC cannot be advised as first line investigation. Same day CTC on failed colonoscopy is desirable but difficult to achieve. Royal College of Radiology UK guidelines advise CTC in adults who fail colonoscopy. 

Experience of endoscopist is known factor for CIR, minimum criteria set out by JAG for certification is 200 colonoscopies. The CIR increases by raising the number of colonoscopies (12). The results also explain slightly higher failure rate among surgeons due to lower procedure volume. The higher the procedure volume the higher is the success rate (13). 

Other alternatives like double balloon endoscopy has also been tested for failed colonoscopy with success rate of 88 to 97 % depending on expertise of endoscopist (14-17). However, its cost and availability is still a drawback.

Interestingly in this study we picked up that high BMI had less failure rate. Which can help to attempt colonoscopy in obese patient with confidence. Possible explanation is extra colonic fat keep the colon straighter. 

Our study also highlights that due to expertise and training of screening endoscopist, very few of them fail. Which means that a repeat procedure for failed colonoscopy should be referred to a more experienced endoscopist for second attempt.

Our study has limitations that we acknowledge. First, it is a retrospective review of cohort with limited numbers. Second, the wide variation in experience of the endoscopist performing the first colonoscopy may or may not have contributed to higher incompletion rates. 

Finally, our institution uses scope guide only for training purposes. Availably of scope guide for all the cases may prevent looping or early correction of looping, leading to less chance of failure. 

Our study highlights almost same issues as previous studies. We recommend that although most of the patients’ factors cannot change, to avoid failure in patients with multiple factors i.e low BMI, female and with pervious pelvic surgeries, the procedure should be done only with scope guide. 

A repeat procedure should be done only for senior endoscopist, especially screening endoscopist, which have more vigorous training and expertise. 

Wide availability of scope guide in all procedures may decrease failure rate. Other modalities of investigating the colon (CTC, DCBE, balloon enteroscopy) should be reserved for failed re-scope patient only.

## Conflict of interests

The authors declare that they have no conflict of interest.
